# Supplementary material for: Stabilin-1 plays a protective role against Listeria monocytogenes infection through the regulation of cytokine and chemokine production and immune cell recruitment
Source: Virulence. 2021 Aug 10;12(1):2088–103. doi: 10.1080/21505594.2021.1958606 (PMC8366540; doi:10.1080/21505594.2021.1958606)
Supplement: Supplemental Material [file KVIR_A_1958606_SM3893.zip › Figures_STAB1_2021_Revision_1 (1).docx]

**Figure S1.** STAB-1 does not have a role in bacterial internalization by non-phagocytic cells.

**Figure S2.** STAB-1 KO mice do not have significant defect on cytokine production.

**Figure S3.** Gating strategy applied on cells recovered from the spleen - myeloid cells (CD11b^int/hi^), neutrophils (CD11b^hi^Ly6G^hi^CD11C^-^), dendritic cells (CD11b^int^CD11c^hi^Ly6G^-^), macrophages (CD11b^hi^Ly6C^int/-^Ly6G^-^CD11c^lo^) and inflammatory monocytes (CD11b^hi^Ly6C^hi^Ly6G^-^ CD11c^lo^), and from the peritoneum - myeloid cells (CD45^+^CD11b^int/hi^), neutrophils (CD45^+^CD11b^hi^Ly6G^hi^CD11C^-^), dendritic cells (CD45^+^CD11b^int^CD11c^hi^Ly6G^-^), macrophages (CD45^+^CD11b^hi^Ly6C^int/-^Ly6G^-^CD11c^lo^) and inflammatory monocytes (CD45^+^CD11b^hi^Ly6C^hi^Ly6G^-^ CD11c^lo^) of non-infected WT mice.

**Figure S4.** Non-infected STAB-1 KO BMDMs are more pro-inflammatory than WT BMDMs.

**Figure S5.** STAB-1 KO mice do not have significant defect on myeloid cell population.

**Table S1** Primers
